# Demographic Variables for Wild Asian Elephants Using Longitudinal Observations

**DOI:** 10.1371/journal.pone.0082788

**Published:** 2013-12-20

**Authors:** Shermin de Silva, C. Elizabeth Webber, U. S. Weerathunga, T. V. Pushpakumara, Devaka K. Weerakoon, George Wittemyer

**Affiliations:** 1 Colorado State University, Department of Fish, Wildlife and Conservation Biology, Fort Collins, Colorado, United States of America; 2 Elephant, Forest and Environment Conservation Trust, Colombo, Sri Lanka; 3 Stirling University, Stirling, Scotland, United Kingdom; 4 University of Colombo, Colombo, Sri Lanka; 5 Trunks & Leaves Inc., Somerville, Massachusetts, United States of America; SUNY College of Environmental Science and Forestry, United States of America

## Abstract

Detailed demographic data on wild Asian elephants have been difficult to collect due to habitat characteristics of much of the species’ remaining range. Such data, however, are critical for understanding and modeling population processes in this endangered species. We present data from six years of an ongoing study of Asian elephants (*Elephas maximus*) in Uda Walawe National Park, Sri Lanka. This relatively undisturbed population numbering over one thousand elephants is individually monitored, providing cohort-based information on mortality and reproduction. Reproduction was seasonal, such that most births occurred during the long inter-monsoon dry season and peaked in May. During the study, the average age at first reproduction was 13.4 years and the 50^th^ percentile inter-birth interval was approximately 6 years. Birth sex ratios did not deviate significantly from parity. Fecundity was relatively stable throughout the observed reproductive life of an individual (ages 11–60), averaging between 0.13–0.17 female offspring per individual per year. Mortalities and injuries based on carcasses and disappearances showed that males were significantly more likely than females to be killed or injured through anthropogenic activity. Overall, however, most observed injuries did not appear to be fatal. This population exhibits higher fecundity and density relative to published estimates on other Asian elephant populations, possibly enhanced by present range constriction. Understanding the factors responsible for these demographic dynamics can shed insight on the future needs of this elephant population, with probable parallels to other populations in similar settings.

## Introduction

Individual-based long-term monitoring studies that build on detailed longitudinal life histories are important for understanding social, demographic, and ecological processes [1]. This is particularly relevant for large-bodied species with extended longevity, such as elephants, which mature and reproduce relatively slowly. Long-term studies of African elephants (*Loxodonta africana*) have documented similarities and disparities in the dynamics of elephant populations at different locations, as well as the demographic responses of those undergoing stress through drought and poaching [2]–[5]. However, less is known about the life-history traits of Asian elephants (*Elphas maximus*) in the wild. Detailed demographic data are critical for establishing and predicting population trends of these keystone species as well as informing management decisions [6], [7]. Moreover because elephants are key players in structuring entire habitats, their population dynamics are likely to have cascading effects throughout ecosystems [8]–[10].

While there are extensive efforts to breed Asian elephants in captivity, and corresponding investment in understanding reproductive physiology in captive or semi-captive settings [11]–[14], estimates of life-history traits derived from captive populations may not accurately reflect those in natural populations for a range of reasons including differences in genetic diversity, resource availability, stress levels, and human demands for labor. Documentation of reproductive and demographic variables for wild populations by contrast are scarce [13], [15]–[17]. Life-history variables for Asian elephants in the wild are challenging to document due to their longevity and relatively slow rates of reproduction, compounded by the inaccessibility of their habitat and tendency to be cryptic in the presence of humans. Moreover the amount of area available to Asian elephants continues to decline compared to their historical range, with the result that many populations are small, fragmented, disturbed and difficult to observe [18]–[20]. Together these factors make it difficult to obtain data from sufficient numbers of individuals as to make statistically adequate estimates. We report vital rates for a relatively large and well-habituated wild population of elephants in Sri Lanka. A subset of females and calves in this population (N = 419) was observed systematically over six years, providing individual-based data on reproductive trends, injuries, and mortalities. We also discuss comparable data for other wild and captive populations where they are available. These estimates are intended to fill a gap in our current knowledge of demographic variables in Asian elephants outside captivity, which are necessary if we are to manage and conserve both elephants and their wilderness.

## Methods

### Ethics Statement

This research was approved by the Institutional Animal Care and Use Committees of the University of Pennsylvania (protocol no. 801295) and Colorado State University (protocol no. 11–2816A). Personnel of the Department of Wildlife Conservation provided information on carcasses, and permission to conduct this research was granted by its Research Committee.

### Study Site

Uda Walawe National Park (UWNP) is located between latitudes 6°25′–6°34′N and longitudes 80°46′–81°00′E in south-central Sri Lanka, very close to the equator. It features large and small man-made reservoirs that provide water for human and wildlife consumption. Other common herbivores include domestic and feral water buffalo (*Bubalus bubalis*) and spotted deer (*Axis axis*). The largest carnivores include the leopard (*Panthera pardus cotiya*), jackal (*Canis aureus naria*), and mugger crocodile (*Crocodylus palustris*), but it is unknown whether they pose any threat to elephants. UWNP has a seasonal, mesic environment, with nearly constant day length throughout the year. The region receives two annual monsoons, generally occurring from October–December and March-April. Rainfall averaged 1514±388 mm annually between 2007–2012 (measured by a standard US weather bureau rain gauge, and calibrated according to a national weather station in Rathnapura). Dynamic and persistent changes in vegetation due to fire and invasive *Lantana camara* (an introduced species of herbaceous flowering plant) during the course of the study converted the majority of tall grassland (the dominant vegetation cover on >50% of the study area at the outset) to mixed scrubland. Over 2/3 of the reserve perimeter is currently electric fenced, but wildlife movement in and out of the park remains possible.

### Long-term Monitoring

**Figure 1:**
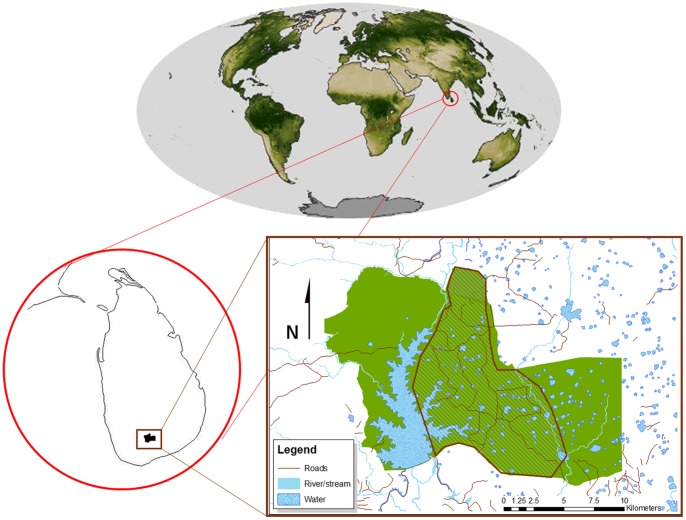
Uda Walawe National Park. The study was conducted along the road network in the middle portion of the park, represented by the hashed lines (<100 Km^2^). Images sources: NASA Earth Observatory (http://earthobservatory.nasa.gov), the Department of Wildlife Conservation, Sri Lanka, and the Center for Conservation Research, Sri Lanka.

UWNP hosts an estimated population of between 800 and 1160 elephants that remains relatively stable over multiple years, although individuals varied in the frequency with which they were seen at different times of year [17]. A pilot study spanning 20 days was conducted in August 2005 and the remaining data were collected between June 2006-December 2012 as part of an ongoing study. Observations inside the park were conducted between 0630–1800 h, three days per week on average by vehicle along the road network in approximately the middle third of the park (Figure 1). Effort was distributed evenly year-round and across years except January–April 2008 for which data were not available due to closure of the park during a period of political unrest [17].

Photographic and demographic records for known individuals were maintained. Individuals were identified using natural features of the ears and other physical attributes [21] (see also Table S1 and Figures S1–S2). To date we have individually-identified 409 individuals in the subadult or adult male size classes, along with 465 individuals in the subadult or adult female size classes. Known adults for whom the interval between sightings was <1 year on average were recorded as dead either when they were observed to have died (N = 3) or when they were not seen for two or more years (N = 10). Mortalities of nursing calves were inferred if mothers were seen without them on three or more occasions and/or mothers exhibited reduction in breast size after the calf went missing (N = 9). Data on mortalities were additionally collected from opportunistic investigations of carcasses (N = 15) inside and within 5 km of the park boundaries and corridors whenever such deaths were reported. Causes of death were inferred whenever possible either through direct observation (such as gunshots and other physical injuries) or interviews with personnel from the Department of Wildlife Conservation (DWC) who conducted investigations.

### Age Estimation

Estimated birth dates for individuals in the study population were assigned an accuracy rating of 1–5. Accuracy 1 indicates calves that were seen within one month of birth, 2 indicates those seen within two months, 3 indicates those for whom the year was known but month was not, 4 indicates those for whom the birth year was estimated by size relative to the height of an adult female [17], and accuracy 5 indicates those who were already adults at the time of the study and, as a result, for whom birth year was approximated according to physical attributes (Table 1). Over the entire monitored population (N = 419), 52 calves were seen within a month of birth (assigned an accuracy rating of 1), 32 within two months (rated 2), 38 within a year (rated 3), 49 were aged based on size at an older age (rated 4), and the remaining 248 were adults when first identified (rated 5). Adult females were defined as those having had at least one calf rather than according to their estimated age class [17]. Age classes 0–13 were known with a high degree of accuracy (1–4) based on the observation of growth in known-aged individuals who were five years old or less at the start of the study. In this paper we focus on demographic data for females and their dependent young, but provide information on injuries and mortalities for males as well.

**Table 1:** Visual cues used for adult female age-class assignment.

Age class	Gross attributes*
11–15	Rounded head shape and ears, square body in profile, rounded pelvic region/base of tail (rump). Primiparous females begin to show breast development during pregnancy.
16–30	Lengthening of back and ears to assume more rectangular shape, head retains similar size in proportion to body as in sub-adults. Squaring of pelvic/base of tail region, protrusion of spinal, pelvic and basal tail bones, (generally) well-developed breasts. One or more dependent calves evident.
31–50	Hollowing of cheek cavity and forehead above brow and temples, enlargement of head in proportion to body size particularly of domes on upper cranium (not always evident), splaying of toes.
>50	Loss of body mass especially around skull and spine, protrusion of cheek bones and temple, hollow cheeks when mouth is closed, (i.e. when not chewing, indicative of lost teeth). May begin to show reduction in breast size unrelated to loss of a calf.

* The apparent age of an animal also varies with body condition, weight loss making it appear older. Ear folds and depigmentation are not reliable indicators of age class. If these visual attributes are used in other populations, corresponding ages should be assessed relative to individuals whose age can be confidently estimated, and after repeated observations of the same individuals to account for changes in condition.

**Figure 2:**
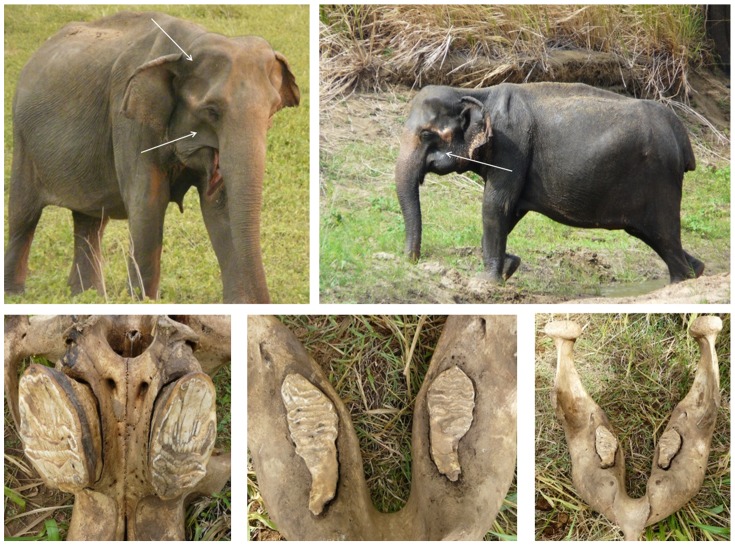
Female no. 33 (‘Tailless’), reference female for oldest age class. This unique female, who was distinctly identifiable even after death due to her tail which was broken at the base, was among the oldest individuals observed in this population. Top panels: Tailless in 2008 exhibits the sunken forehead and cheeks as well as reduction in breast mass characterizing the >60 yr age class. Bottom panels: both upper and lower molars exhibit severe wear with no signs of additional teeth to emerge.

Among adults, age classification was established from (1) comparison with individuals aged post-death (Figure 2) and (2) age-specific physical attributes described elsewhere [21], [22] and developed in this study, including visual assessments of head morphology, body development, and size relative to one another as well as the height and length of our observation vehicle or trees when they stood next to it. Males may continue growing throughout their lives, but female gain little height once they are of reproductive age. A mature adult female in the oldest age class typically had a back length of ∼2.3 m and shoulder height of ∼2.1 m in our population, but high variability in body size limited the utility of using such characteristics without additional calibration. Females and the majority of males in this population are tusk-less, thus tusk growth could not be used as an indicator of age as in other populations. Molar assessments [23] were also impractical as most carcasses were never found. We anchored age classes relative to one of the oldest adult females (60+years) whose jaw was recovered after death (Figure 2). Because estimates based on these criteria are coarse, we grouped females aged 10–20 yrs into 5-year age classes and those over the age of 20 into 10-year age classes.

### Fecundity, Primiparity, and Inter-birth Intervals

Because the duration of the study was short relative to the developmental trajectory of elephants, we offer multiple estimates of vital rates with varying degrees of accuracy. We calculated age-specific fecundity over all females aged 0 to over 60 for whom ages could be estimated (N = 280). We first estimated the age at primiparity (first birth) using only those females known with certainty to have had their first calves during the study period, for whom data are most accurate. We then used standard lifetable approaches to derive the hazard function of age-specific primiparity among all identified females born between 1995–2003 (N = 34).

We estimated the inter-birth intervals (IBI) using multiple cohorts representing decreasing levels of accuracy but increasing sample sizes: (a) females for whom two successive births were observed within two months of the event (N = 13) (b) those for whom two successive births were recorded any time during the study period (N = 27), and (b) those for whom one birth was estimated to have occurred within 2 years of the start of the study and the second birth was observed (N = 37). We then present the hazard function for IBI calculated using the cohort of females that were of reproductive age between 2005–2012 (N = 92), excluding individuals who died, were seen infrequently, were over the age of 60, or were not parous before 2010.

Results are reported as averages ± estimated standard errors except where noted, and samples sizes are as specified. All statistical analyses were conducted in R v. 2.15 [24].

## Results

**Figure 3:**
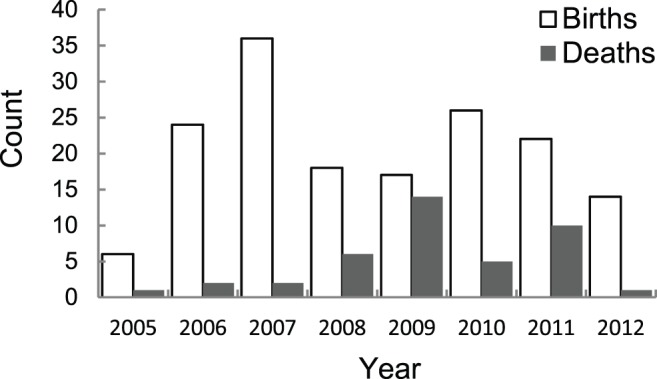
Annual totals of recorded births and deaths. Although there are fewer mortalities than births observed, this should not be taken to indicate a growing population. Most carcasses go undetected due to the dense cover. Moreover chances of detection are likely to be even lower for individuals near the age of dispersal, because disappearance through death vs. dispersal are not distinguishable in this study.

**Figure 4:**
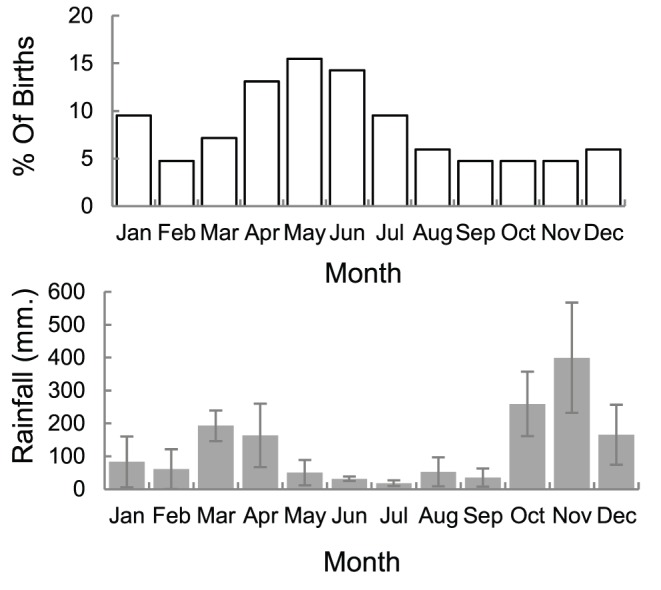
Births and rainfall periods. Top: Frequency of births by month for those accurate to within two months (N = 84). Bottom: Average monthly rainfall. Error bars indicate inter-annual variation (SE).

Between 2005–2012, 163 births to 131 unique females (∼28% of those that were identified) and 41 deaths were recorded as described in the methods (Figure 3). Of the calves for whom birth month was known to within two months or less (n = 84) most were born from April–July, which predominantly falls within the dry season. Accounts in the literature report gestation periods ranging from 19–23 months [13], [16]. Assuming 22 months of gestation, conceptions may peak toward the end of the five-month dry season, out of phase with the period of highest primary productivity from October–January (Figure 4).

**Figure 5:**
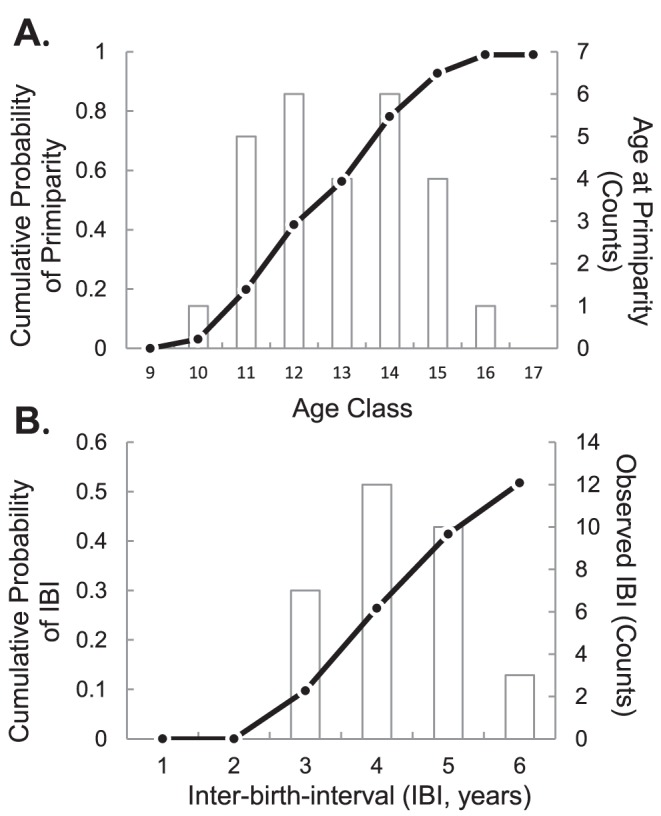
Estimated (a) ages at primiparity and (b) inter-birth intervals (IBI). Curves represent the cumulative probability that a female gives birth to her first calf at a particular age (a), or exhibits an IBI of a specified length (b). Histograms show counts of observed ages at primiparity and birth intervals respectively. Data are included as Tables S2 & S3.

Twenty-one females were known with certainty to have had their first calves during this study period. The average age at primiparity for these individuals was estimated to be 13.4±2.4 years. Figure 5a shows the cumulative probability of reproducing based on an expanded cohort of 33 individuals born between 1995–2003. The 50^th^ percentile for these estimates also fell at 13 years. The mean age at first conception was lower at Uda Walawe than those reported for some populations in India, but slightly higher than other populations in Sri Lanka for which these variables were approximated based on age structure (Table 2).

**Table 2:** A comparison of demographic variables across populations.

Location & type	Period of study	Age at 1st conception (yrs)	Age at senescence	Mean Inter-birth interval (yrs)	Birth sexratio (F/M)	Adult sexratio (F/M)	Fecundity	Elephant density(per km^2^)	Sources
Timber camps(Semi-captive, Myanmar)	1925–1999	5.3	53	5	0.93	n/a	–	n/a	[26], [31]
Forest camps, Tamilnadu(Semi-captive; India)	1925–1996	11–13	63	10.5	1.18	n/a	0.095–0.155	n/a	[27]
Biligirirangans-Nilgris (Wild; India)	1981–1994	14–15	–	4.5–4.7		5	0.21	0.53–2.0	[16], [41]–[42]
Bilgiri Rangaswamy Tiger Temple Reserve (Wild; India)	2009–2010	–	–	–	–	4.1	–	1.7	[43]
Ruhuna-Yala & Southeast (Wild; Sri Lanka)	1960–1990	∼10*	–	4.0–6.5*	–	1.19	–	0.25	[15], [44]–45
Gal Oya National Park (Sri Lanka)	1975–1976	–	–	–	–	2.85	–	–	[32]
Amparai Sanctuary (Sri Lanka)	1975–1976	–	–	–	–	1.66	–	–	[32]
Wasgamuwa (Wild; Sri Lanka)	1980–1982	∼10*	–	4.0*	–	–	–	0.5	[46]
Uda Walawe (Wild; Sri Lanka)	2006–2012	11	∼60	3.9	1.28	1.18	0.157	>3.5	[17], this study

Fecundity is given in terms of the average number of female offspring per breeding female per year. Adult sex ratios for captive populations are not given since wild-caught individuals are included therein.

* Inferred by Sukumar (2003) from age structure in original studies.

Estimates of inter-birth interval (IBI) are summarized in Figure 5b and Table 3. At least 26 females (16% of individuals over the age of 10 at the start of the study) were not observed to have had any new calves during the period of the study. Of these 11 had died by 2012, and the other 15 were estimated to be over the age of 60. Of those that died, 9 were estimated to be over 65 years old and appeared to be reproductively senescent. Among the 92 females sampled, 65 gave birth only once between 2005–2012, while 27 had more than one calf. All five females for whom the IBI was <40 months lost the first of their two calves within a year or less. When these females were excluded, the IBI for the remaining 22 females observed to give birth twice during the study averaged 50.7±7.5 months. The 50^th^ percentile of the cumulative probability of IBI based on the cohort of 92 females was approximately 6 years (72 months). The five who lost their calves in contrast had intervals of 26–38 months.

**Table 3:** Estimates of inter-birth-intervals with datasets ordered by decreasing accuracy.

Sample criterion	Time Interval (span)	N females	N births		Avg. IBI (months)
Both births recorded within 2 months	2006–2012 (∼6.5 yrs)	13		26	47.5±11.6
Both births occurring after 2005, regardless of accuracy	2006–2012 (7 yrs)	27		54	47.2±10.3
Females with calves <3 in 2005	2003–2012 (10 yrs)	37		78	53.3±14.5

**Figure 6:**
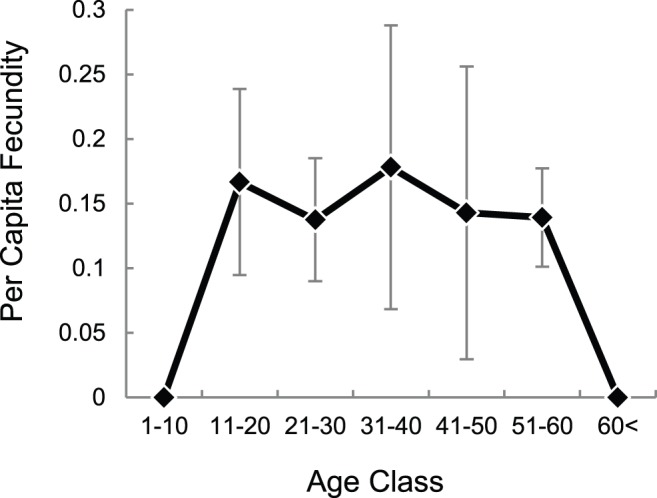
Fecundity averaged over births observed from 2006–2012 for N = 280 females. The average ranges between 0.13–0.17 female offspring per individual per year. Data are provided in Table S4.

Out of the 84 calves with birth dates accurate to two months, the sex was determined for 73 (87%, Table 4). The overall ratio of females to males was 1.28±0.30 (standard error was estimated as in Skalski et al., 2005 [25] without the finite population correction as the number of calves in the entire population is unknown). This did not indicate a significant bias (binomial test, p = 0.34). Average fecundity in terms of the number of female calves produced by each age class per capita per year was relatively stable across age classes (Figure 6), though the actual number of female offspring per age class fluctuated across years. Table 5 shows the proportion of individuals in each age class from the sample of identified individuals alive at the end of 2012. While there are equal numbers of calves of both sexes aged 0–2 yrs, there are consistently fewer males in subsequent non-adult age classes.

**Table 4:** Sex of calves when births were observed within 2 months and sex was determined.

Age class of mother	Females	Males
11–15	6	13
16–20	4	0
21–30	5	8
31–40	14	4
41–50	9	2
50<	3	5
Total	41	32

**Table 5:** Composition of cow-calf units consisting of known individuals alive at the end of 2012.

Age class	Female	(%)	Male
0–2	13	(4.7)	13
3–5	31	(11.3)	29
6–10	57	(20.8)	37
11–15	26	(9.5)	20
16–20	17	(6.2)	–
21–30	31	(11.3)	–
31–40	34	(12.4)	–
41–50	28	(10.2)	–
51–60	21	(7.7)	–
>60	15	(5.8)	–
Total	274		99

Males are excluded here after the age of dispersal because of the lower confidence in age classification, therefore the proportion represented by each age class is presented for females only.

Calves under the age of four repeatedly seen isolated or without their mothers exhibited signs of malnourishment such as loss of body mass, distended abdomen, and stunted growth. Out of five such cases, two were confirmed to have died (estimated at ages <1 year and 3 years), two disappeared (aged approximately 2 years and 4 years). The fifth lost his mother at the age of 3 and survives to the age of 7 by the end of 2012, but shows no gain in height or body size. In contrast, a male that lost its mother at the age of 7 showed a brief decline in health but recovered, exhibited normal growth and survives today having reached the age of 11 by the end of the reported study period.

**Figure 7:**
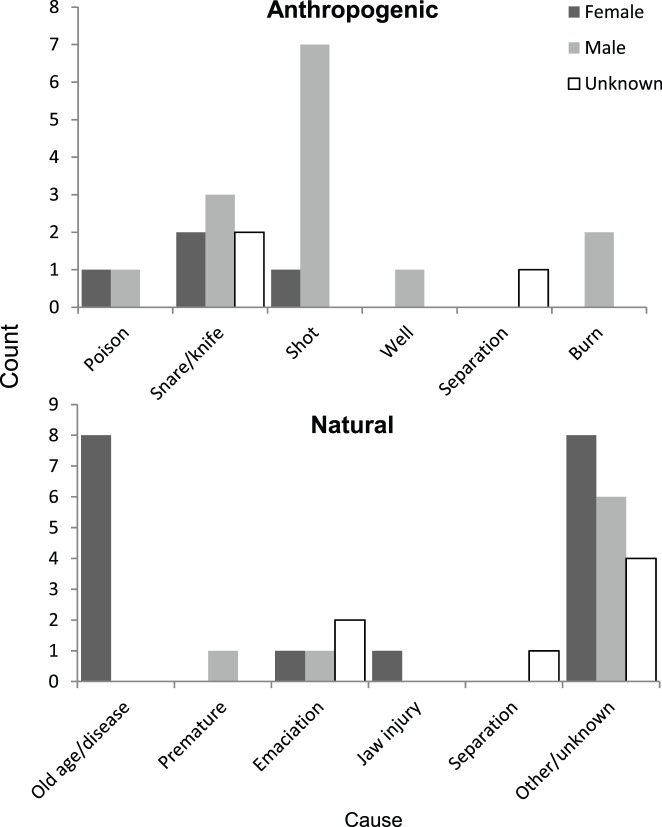
Injuries and mortalities by sex and cause. Anthropogenic causes - *Poison*: ingestion of toxic substance, usually hidden in food items such as pumpkins. *Snare/knife*: wounds that encircle or cut into an appendage from wire or rope. *Shot*: gunshots and swellings from gunshots. *Well*: abandoned water wells in which animals can fall. *Separation*: death of a calf due to death or disappearance of the mother after a human-caused injury. *Burn*: skin lesions caused by fire. Natural causes – *Old age/disease*: disappearance or confirmed death within the oldest age class, or known illness. *Premature*: calf born prematurely. *Emaciation*: extreme loss of body mass. *Jaw injury*: swelling or distortion of the lower jaw. *Separation*: separation of a calf from the mother without any sign of injury to the mother. *Other/unknown*: unknown circumstances, here grouped with ‘natural’ causes but human influence cannot be excluded. Includes the cases of isolated calves, where reasons for the absence of the mother were not known.

Injuries seen on males and females were frequently caused by humans (Figure 7). Wounds from snares and gunshots were the most common, with several animals exhibiting healed wounds from prior years. Out of 24 fresh injuries observed on live animals due to either human or natural causes, only five were later confirmed or suspected to have died (i.e. through finding the carcass or disappearance). Figure 7 summarizes mortalities by sex and cause (see also Figure S3 for breakdowns by year and age class). For injuries and mortalities in which causes were inferred (N = 34), males and females were equally represented and those thought to be natural vs. anthropogenically influenced were nearly evenly split (47% and 53% respectively). However, males were significantly more likely than females to die or exhibit injuries through human activity (Fisher’s exact test, two-tailed p<0.025).

## Discussion

While approximately a quarter to a third of the living Asian elephants may be in captive or semi-captive situations that are closely monitored [14], [16], [26]–[29], very little is known about the remaining wild populations that are scattered throughout South and South East Asia. The disparity has tended to reinforce a misperception among the public in non-range states that Asian elephants are a nearly domesticated species with little hope of survival in the wild. This distortion must be corrected if wild populations are to continue performing their irreplaceable ecological roles as seed dispersers and ecosystem engineers [8]–[10] because public awareness *ex situ* is necessary to support conservation efforts *in situ*. In fact, the opposite may be true. It is well-documented that elephants do not breed as well in captivity as they appear to do in the wild, tending have lower fecundity, higher calf mortality, and male-biased birth sex ratios [27]–[30]. Though a few females born in captivity may reach sexual maturity at a much younger age than their wild counterparts (Table 2), the tendency for many individuals to reproduce later and reach senescence earlier in captivity [31] may shorten the lifetime reproductive output an individual. Thus even in the most productive captive or semi-captive situations, breeding may not keep up with the demand. As a result wild-caught animals continue to supplement the demand driven by tourism or labor needs, and the off-take may actually threaten the integrity of wild populations [14].

Estimates of demographic variables for Asian elephants from UWNP can serve as a benchmark dataset in the sense that they are derived from a closely-monitored wild population. In this study we provide data from detailed longitudinal observations on well-habituated animals. The presence of very old individuals in the population also suggests elephants at this location may reach their full reproductive potential. This provides a valuable opportunity to examine the natural reproductive dynamics of an elephant population, even with the constraints imposed by electric fencing and anthropogenic disturbance in and around the protected area. However, as the study is of relatively short duration and estimates involve small sample sizes, findings may not be sufficiently general. Data from captivity and from the wild may usefully complement one another and yield fresh insights by comparison. Nevertheless, one cannot and should not substitute one for the other. We hope these data may be useful for captive breeding programs in evaluating the reproductive health of their animals, and also to inform conservation efforts in the wild by providing concrete estimates of vital rates on which to base population models.

### Reproduction and Growth

Although the density of elephants in UWNP is very high compared to those reported in the literature (Table 2), the age at first reproduction and inter-birth intervals we observed indicate a productive population. The annual peak in births toward onset of the dry season (far in advance of the monsoon) is similar to patterns reported for captive elephants in Myanmar and India [16], [27], [30]. In the Myanmar timber camps, calves born during the peak birthing months had a lower probability of dying before the age of 5 [30]. However, as the same months also corresponded to resting rather than working periods for the mothers, seasonal and anthropogenic effects on births as well as mortalities were difficult to disentangle. Because this confound does not hold for the wild population in our study, the cycles are more likely to actually reflect ecological influences and it would be of interest to see in future whether there is a similar effect on calf survival. However, these findings contrast to what was observed in Gal Oya, Sri Lanka, during a 22 month study in which an apparent birth peak was observed toward the end of the dry season in the first year, but not the second [32]. There appears to be some variability across populations therefore in the degree of coupling between births and seasonality, which would benefit from further exploration.

The median age at first reproduction (as opposed to conception) was approximately 13 years. Fecundity remained constant from the age at first reproduction until around the age of 60, from which point no female in our study was observed to give birth. Note however that because there are fewer individuals in this age class (Table 5) and the relatively short duration of the study, this does not necessarily indicate that individuals stop reproducing beyond this age. Birth sex ratios did not show significant bias, although there was a slight tendency to observe more females than males compared to records from captive or semi-captive populations (Table 2). At least two (non-mutually exclusive) explanations are possible. Females in the wild may be in poorer condition than those in captivity and thus more likely to have female offspring, in conformity with the Trivers-Willard hypothesis [47]. When broken down by the age class of the mother (Table 4), there seems to be a tendency for primiparous females to have more males compared to females in their peak reproductive years. Alternatively, early male-biased mortality may go undetected in the wild, where there is a lag between birth and observation. Table 5, however, suggests this is not the case. Larger sample sizes are needed to evaluate these effects.

Estimates of average IBI derived from the most accurate data (calves seen within two months of birth, and births estimated to have occurred during the study period) are in close agreement despite doubling the sample size (Table 3). Both are slightly lower than other published estimates. These estimate may be biased low in that females with long birth intervals were not well-represented in this study and the subsets of known individuals who gave birth twice are small (Table 3). When we include calves born shortly before the study period, the estimated IBI rises by three months to the more typically reported range of 4–6.5 years (Table 2). The 50^th^ percentile IBI value (Figure 5b) falls at 6 years. It is interesting to observe that the average IBI also increased after excluding those females who lost their calf within the first year (despite a reduction in sample size). Taken together, these observations suggest that ignoring calf mortalities increases the apparent IBI. Published estimates of IBI for wild Asian elephant populations are typically inferences from cross-sections of the age structure of living offspring seen during short-term observations. Such estimates would miss calf mortality as well as individuals with longer birth intervals, where two consecutive offspring are not simultaneously visible. Additional years of data on the Uda Walawe population would enable the IBI to be estimated with greater confidence.

The individuals for whom the most complete and accurate data were collected also happened to be those occupying central areas of the park, largely buffered from human disturbance aside from tourism. These data potentially understate human impacts affecting the broader population. There is a high degree of seasonal turnover within the park [17]. Those occupying more peripheral areas and ranging outside the park, where observations are more difficult and where they are more exposed to human activity, may exhibit demographic differences.

Calves in this population were usually weaned as soon as a sibling was born, around the age of four. But a few may nurse up to the age of five or more in the absence of a younger sibling. Some females were observed nursing two calves simultaneously, and one exceptional female permitted all three of her calves to nurse in close succession. The older calf was approximately seven or eight years old. Our observations based on those rare cases in which calves appear to have been orphaned before the age of 4 suggest that milk is important for normal growth at least up to this age, even though they do consume herbaceous vegetation. This is similar to what was found using the largest demographic dataset on semi-captive elephants. A recent study compiling multiple decades of data on elephants from logging camps in Myanmar reported that while the leading cause of death among calves under the age of 5 was accidents (42.4%) the second most common cause was calf weakness due to maternal agalactica, or lack of milk, and general weakness of the neonate (26.3%) [26]. Maternal condition has been shown to affect the duration of lactation in several species of large bodied mammals, with young typically weaned when they attain four times their birth weight [33]. In the absence of predation, both in the wild and semi-captivity, maternal condition may play an important role in regulating generation time in Asian elephants via its effect on calf growth and survival.

### Injuries and Mortalities

The majority of sightings involving injuries did not appear fatal though several adult males habitually appeared in Uda Walawe exhibiting gunshot wounds in the legs, side, or pelvic region. One adult male in the 31–50 age class appeared in 2012 with multiple swellings from gunshot wounds to the legs which subsided within a month. Three males including a tusker and one (pregnant) female were confirmed to have died from gunshot wounds (Figure 7 and S3). Anthropogenic activity was evident in 76% of the injuries and mortalities in males but only 29% in females. This tendency for more males than females to exhibit signs of conflict with humans is similar to what is observed in southern India where 49% of male mortality was attributed to poaching or conflict, compared to 12% for females [16]. The majority of shooting incidents are likely due to crop raiding events, and perhaps intended to deter rather kill the animal. Similarly, burn wounds are caused by defensively throwing or stabbing at animals with clay or cloth flame torches which then adhere to the skin. The tendency of males to be the primary crop raiders is typical of elephants [34], including the sister species *L. africana* which exhibits social differentiation among those that raid habitually and those that do not [35].

Carcasses exhibited two patterns of death other than by physical trauma. Four individuals, all adult females, died after several days of prolonged incapacitation due to unknown causes with no obvious external signs of injury. These were concluded to be predominantly natural, possibly due to malnourishment, old age, or disease etc. In contrast, two adult females and one sub-adult male exhibited signs of sudden death, also without any external indications of injury. Two of these were reported to be poisoned while the third cause was not determined. Our criteria for recording mortality were very conservative. Although the proportion of mortalities relative to births appears low, it is very likely deaths are being under-reported due to the difficulty of detecting carcasses in the dense vegetation as well as our inability distinguish death from dispersal in the intermediate age classes. We therefore present only raw counts of mortalities, rather than estimates of age-specific survivorship in this study. We hope that longitudinal monitoring of known individuals of all age classes will enable such estimates in the future.

### Wider Context

This study demonstrates that wild populations can be productive despite range constriction and the presence of high human population densities. Sri Lanka is estimated to contain approximately 13.5% of the global Asian elephant population, the second largest of all range countries, yet it also contains the third highest density of people per unit area [36], [37]. It is generally thought that scrub and grassland environments can support higher densities of elephants than forest environments [16]. UWNP fits this perspective. In addition, the creation of man-made water sources in and around the park likely augment its carrying capacity. However, an alternate possibility is that the population is exhibiting demographic inertia. As with other slowly-reproducing mammals such as the great apes, the effects of habitat constriction and loss of gene flow may only be visible after substantial time lags [38]–[40]. UWNP is surrounded by agriculture and cultivation, but connected to other patches of scrub and deciduous forest in the north and east. While elephants within the park likely serve as a source population for the region, the ability of these remnant habitat patches to support dispersing individuals is not well known. This raises several important questions to be addressed by future research: how and where are individuals from this population dispersing each year? What other populations are linked to it and what is the extent of gene flow among them? How do dispersing individuals interact with human settlements?

Attention to the demographic dynamics of wild populations is also crucial because elephants influence ecosystem structure through their biotic and abiotic interactions [8]–[10]. Fernando & Pastorini (2011) note that distribution data for Asian elephants are still inadequate, and do not permit “accurate monitoring of distributional changes over time, other than the complete disappearance of an entire population” [36]. Extirpations could be forestalled if the parameters within which populations persist or decline locally could be identified. Failure to monitor them likewise weakens our ability to ensure the persistence of these remnant populations. Unfortunately, if data on distributions is scarce, data on demographics in the wild are almost nonexistent, but for the few studies referenced here. Maintaining longitudinal records on single populations is time-consuming, costly, labor-intensive, logistically challenging and vulnerable to political interference. The health of extant populations and the impacts of human activity on their viability therefore remain poorly known. Nevertheless, concerted efforts must be made to overcome these data gaps and challenges if we are to conserve both elephant populations, and the ecosystems they help to shape.

## Supporting Information

Figure S1 **Examples of features used in individual identification.** The most distinct cues are ear shapes, folds and minor injuries. Cues such as tail length and back are less variable, but reliable. Depigmentation and tail hair quantity/symmetry cues are less reliable.
(PDF)

Figure S2 **Multiple views.** Individuals with asymmetric ears can look different when seen from the right (a) than from the left (b) and be mistaken for two animals. A clear frontal view (c) is therefore preferable before a new ID can be assigned. Veins and/or depigmentation are more clearly visible when wet (a), than when dry and covered in dust therefore these should be secondary cues. Tail length view (d) should show it hanging straight or nearly straight down and is only informative if the tail is unusually long or short. Backbone (e) and forehead profile (a,e) can also be useful at a distance if distinctive.
(PDF)

Figure S3 A–C: Annual confirmed (carcass-based or disappearance of known individual) mortalities by age/sex class. D–E: Annual mortalities and injuries by suspected cause.
(PDF)

Table S1 Features used to identify adult Asian elephants (see Figures S1 & S2).
(PDF)

Table S2 Primiparity. Data presented in Figure 5a.
(PDF)

Table S3 Inter-birth-intervals. Data presented in Figure 5b.
(PDF)

Table S4 Age-specific fecundity per capita. Data presented in Figure 6.
(PDF)
