# Supplementary material for: Demographic Variables for Wild Asian Elephants Using Longitudinal Observations
Source: PLoS One. 2013 Dec 20;8(12):e82788. doi: 10.1371/journal.pone.0082788 (PMC3869725; doi:10.1371/journal.pone.0082788)
Supplement: Figure S1 — Examples of features used in individual identification. The most distinct cues are ear shapes, folds and minor injuries. Cues such as tail length and back are less variable, but reliable. Depigmentation and tail hair quantity/symmetry cues are less reliable. (PDF) [file pone.0082788.s001.pdf]

Figure S1

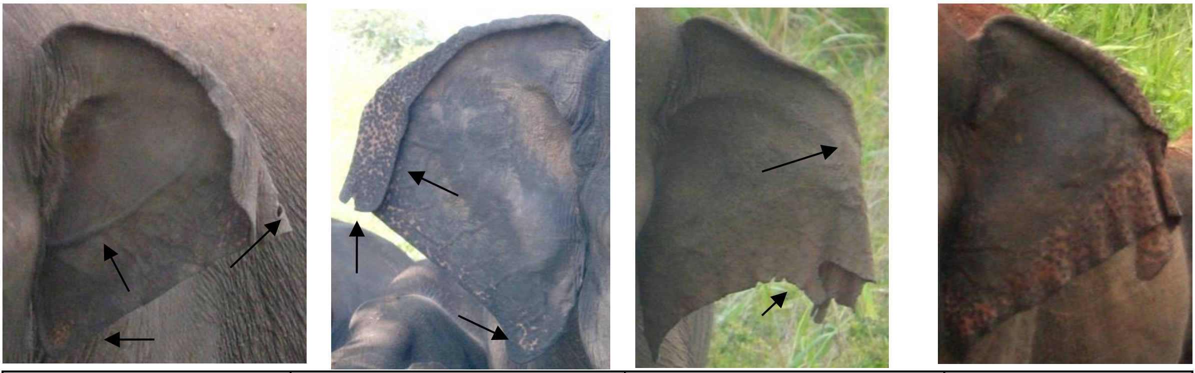

Vein, hole; square earlobe	Notch, 2ndary fold forward; curved earlobe	2ndary fold backward; “finger”	Long ear

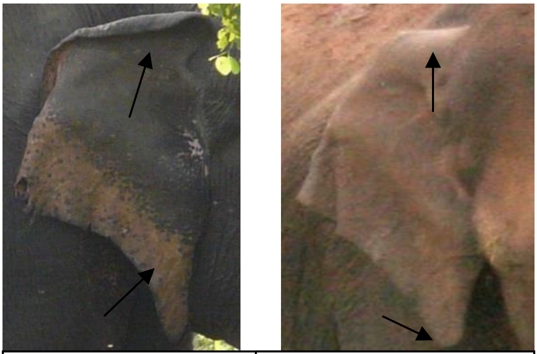

Depigmented, primary fold fwd; long, curved earlobe	Primary fold backward, no depigment; wedge earlobe

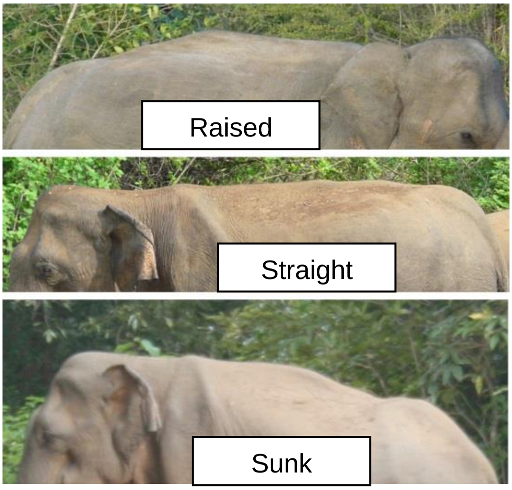

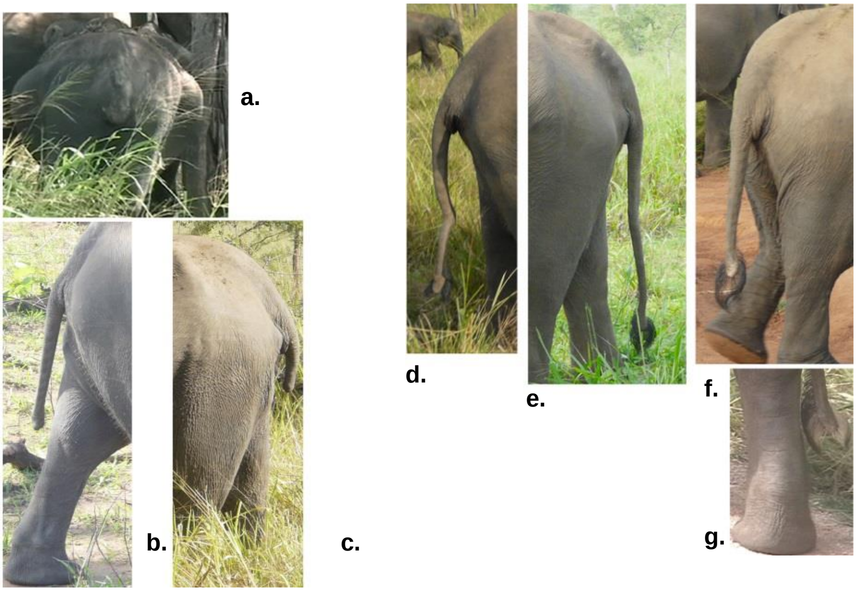

Broken tails (a-c):

a) base
b) tip
c) middle.

Other tail features:

d) crooked
e) long
f) short
g) white hair
