# Supplementary figures and images for: Demographic Variables for Wild Asian Elephants Using Longitudinal Observations
Source: PLoS One. 2013 Dec 20;8(12):e82788. doi: 10.1371/journal.pone.0082788 (PMC3869725; doi:10.1371/journal.pone.0082788)

**Figure S2**

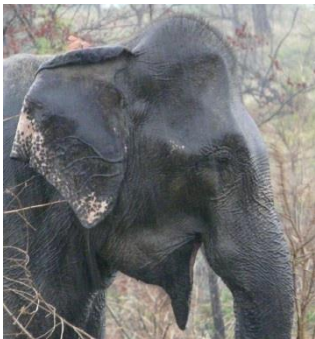

**a. [173] R**

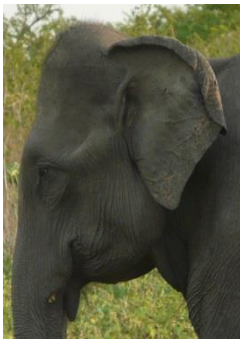

**b. [173] L**

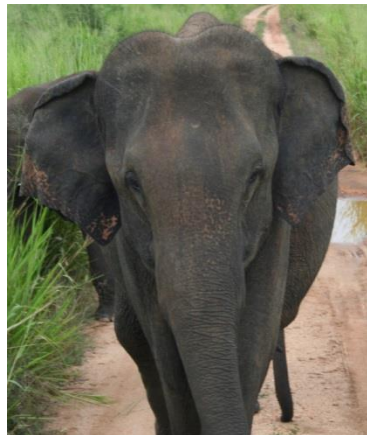

**c. [173] F**

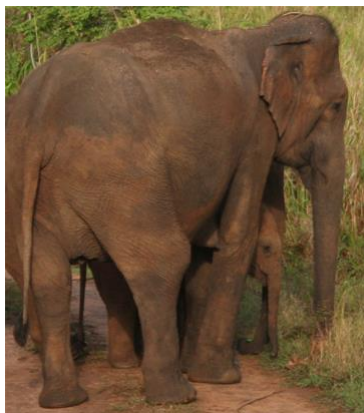

**d. [173] T**

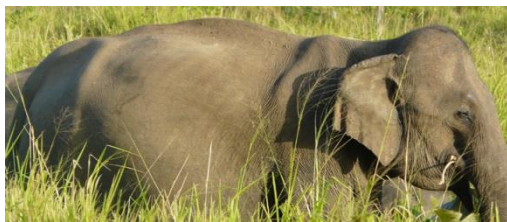

**e. [173] RB**

Supplement: Figure S2 — Multiple views. Individuals with asymmetric ears can look different when seen from the right (a) than from the left (b) and be mistaken for two animals. A clear frontal view (c) is therefore preferable before a new ID can be assigned. Veins and/or depigmentation are more clearly visible when wet (a), than when dry and covered in dust therefore these should be secondary cues. Tail length view (d) should show it hanging straight or nearly straight down and is only informative if the tail is unusually long or short. Backbone (e) and forehead profile (a,e) can also be useful at a distance if distinctive. (PDF) [file pone.0082788.s002.pdf]

Figure S3

**A. Sex unknown**

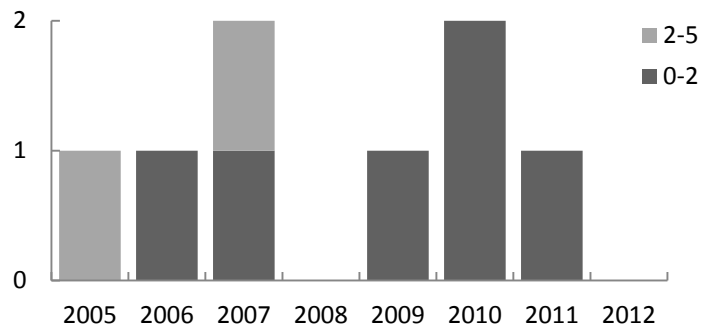

**B. Males**

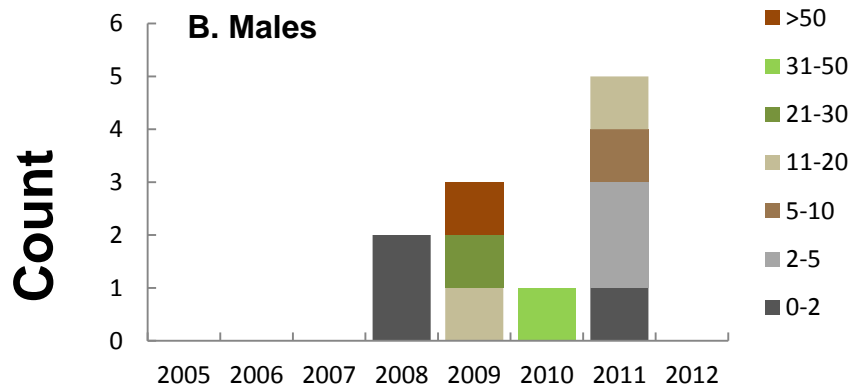

**C. Females**

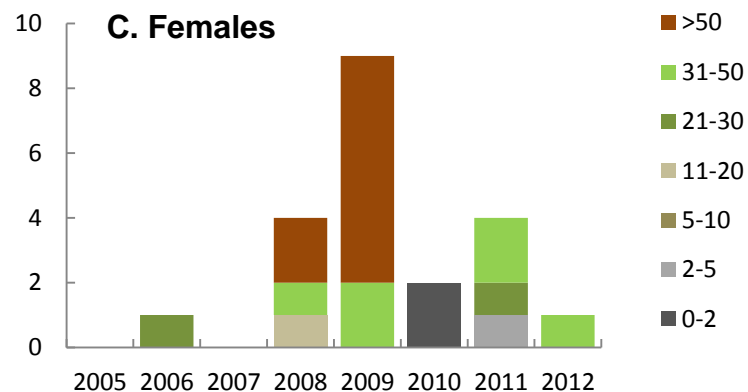

**D. Anthropogenic**

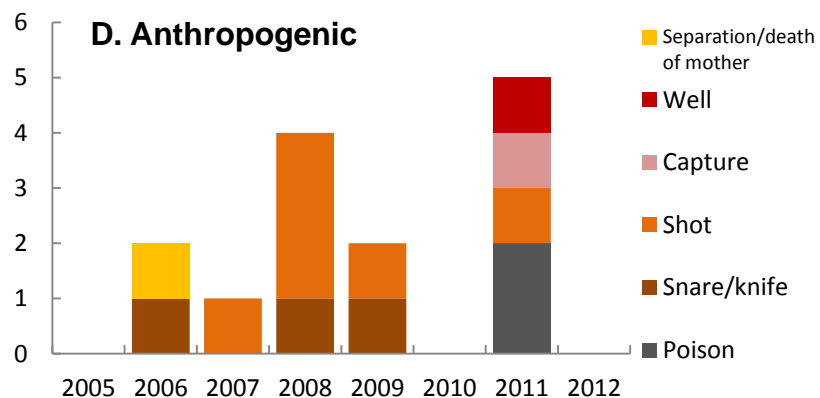

**E. Natural**

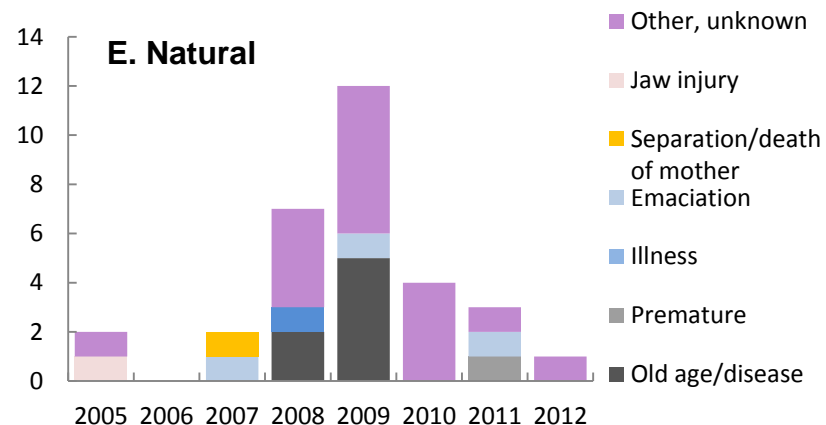

**Year**

Supplement: Figure S3 — A–C: Annual confirmed (carcass-based or disappearance of known individual) mortalities by age/sex class. D–E: Annual mortalities and injuries by suspected cause. (PDF) [file pone.0082788.s003.pdf]
