# Supplementary material for: Demographic Variables for Wild Asian Elephants Using Longitudinal Observations
Source: PLoS One. 2013 Dec 20;8(12):e82788. doi: 10.1371/journal.pone.0082788 (PMC3869725; doi:10.1371/journal.pone.0082788)
Supplement: Table S1 — Features used to identify adult Asian elephants (see Figures S1 & S2). (PDF) [file pone.0082788.s004.pdf]

Table S1 - Features used to identify adult Asian elephants (see Figures S1 & S2).

**Ears:**	**Ear lobes:**	**Tail:**	**Backbone:**	**Other:**
Holes<br>Tears<br>“Fingers”<br>Notches<br><br>Primary fold (forward/backward)<br>Secondary fold (forward/backward)<br><br>Plain (undamaged)<br>Long tips<br>Veins<br>Depigmentation	Long<br>Square<br>Wedge<br>Curve	Long<br>Short<br>White hair<br>Crooked<br><br>Broken (at base, middle, tip)	Straight<br>Raised<br>Sunk	Wounds<br>Growths<br><br>**Face:**<br><br>Forehead profile<br>Depigmentation
