# Supplementary material for: Demographic Variables for Wild Asian Elephants Using Longitudinal Observations
Source: PLoS One. 2013 Dec 20;8(12):e82788. doi: 10.1371/journal.pone.0082788 (PMC3869725; doi:10.1371/journal.pone.0082788)
Supplement: Table S2 — Primiparity. Data presented in Figure 5a. (PDF) [file pone.0082788.s005.pdf]

Table S2 – Primiparity.

Age	No. individuals available	No. primiparous	Cumulative probability of primiparity
9	20	0	0
10	32	1	0.03125
11	29	5	0.198276
12	22	6	0.416928
13	16	4	0.562696
14	12	6	0.781348
15	6	4	0.927116
16	1	1	1
17	0	0	1

Data presented in Figure 5a.
