# Supplementary material for: Demographic Variables for Wild Asian Elephants Using Longitudinal Observations
Source: PLoS One. 2013 Dec 20;8(12):e82788. doi: 10.1371/journal.pone.0082788 (PMC3869725; doi:10.1371/journal.pone.0082788)
Supplement: Table S3 — Inter-birth-intervals. Data presented in Figure 5b. (PDF) [file pone.0082788.s006.pdf]

Table S3 – Inter-birth-Intervals.

Minimum years to next birth	No. potential mothers	No. mothers observed to give birth	Cumulative probability of birth
1	10	0	0
2	10	0	0
3	72	7	0.097222
4	65	12	0.263889
5	49	10	0.414116
6	17	3	0.517507

Data presented in Figure 5b.
