# Supplementary material for: Demographic Variables for Wild Asian Elephants Using Longitudinal Observations
Source: PLoS One. 2013 Dec 20;8(12):e82788. doi: 10.1371/journal.pone.0082788 (PMC3869725; doi:10.1371/journal.pone.0082788)
Supplement: Table S4 — Age-specific fecundity per capita. Data presented in Figure 6. (PDF) [file pone.0082788.s007.pdf]

Table S4 – Age-specific Fecundity.

Age structure	1-10	11-20	21-30	31-40	41-50	51-60	60<
2006	53	26	36	31	27	23	13
2007	69	26	36	30	28	23	13
2008	76	29	36	30	28	23	13
2009	83	32	36	29	29	23	10
2010	91	35	36	29	28	22	6
2011	98	30	30	35	28	22	16
2012	99	38	30	35	28	22	16
Totals	569	216	240	219	196	158	87
Frequency	0.337685	0.12819	0.142433	0.12997	0.11632	0.093769	0.051632
Total fecundity F(x)	0	36	33	39	28	22	0
Per capita, M(x)	0	0.166667	0.1375	0.178082	0.142857	0.139241	0
SD	0	0.071989	0.047544	0.109711	0.113153	0.038061	0

Data presented in Figure 6.
